# Comparing In vitro Protein Aggregation Modelling Using Strategies Relevant to Neuropathologies

**DOI:** 10.1007/s10571-025-01539-z

**Published:** 2025-03-13

**Authors:** André Nadais, Inês Martins, Ana Gabriela Henriques, Diogo Trigo, Odete A. B. da Cruz e Silva

**Affiliations:** Neurosciences and Signaling Group, Department of Medical Sciences, Institute of Biomedicine, University of Aveiro, 3810-193 Aveiro, Portugal

**Keywords:** Alzheimer´s disease, Parkinson´s disease, Aβ1-42 peptide, Protein aggregation, Mitochondrial dysfunction

## Abstract

Protein aggregation is remarkably associated with several neuropathologies, including Alzheimer´s (AD) and Parkinson´s disease (PD). The first is characterized by hyperphosphorylated tau protein and Aβ peptide deposition, thus forming intracellular neurofibrillary tangles and extracellular senile plaques, respectively; while, in PD, α-synuclein aggregates and deposits as Lewy bodies. Considerable research has focused on developing protein aggregation models to be explored as research tools. In the present work, four in vitro models for studying protein aggregation were studied and compared, namely treatment with: the toxic Aβ1-42 peptide, the isoflavone rotenone, the ATP synthase inhibitor oligomycin, and the proteosome inhibitor MG-132. All treatments result in aggregation-relevant events in the human neural SH-SY5Y cell line, but significant model-dependent differences were observed. In terms of promoting aggregate formation, Aβ and MG-132 provoked the greatest effect, but only MG-132 was associated with an increase in HSP-70 chaperone expression. In fact, the type of aggregates formed appear to be dependent on the treatment employed, and supports the hypothesis that Aβ exposure is a relevant AD model, and rotenone is a valid model for PD. Furthermore, the results revealed that protein phosphorylation is relevant to aggregate formation and as expected, tau co-localized to the deposits formed in the Aβ peptide aggregate induction cell model. In summary, different molecular processes, from overall and specific protein aggregation to proteostatic modulation, can be induced by using distinct aggregation modelling strategies, and these can be used to study different protein-aggregation-related processes associated with distinct neuropathologies.

**Supplementary Information:**

The online version contains supplementary material available at 10.1007/s10571-025-01539-z.

## Introduction

In healthy organisms, protein homeostasis is maintained through a series of cellular and organelle-based systems. Under normal conditions, misfolded proteins are targeted for degradation via ubiquitin–proteasome or lysosomes-autophagy systems (Kaushik & Cuervo 2015), and specialized forms of autophagy are also found in the endoplasmic reticulum (ER) and mitochondria (ER-phagy and mitophagy, respectively) (Trigo et al. 2019). Additionally, under proteotoxic stress, the unfolded protein response (UPR) can be activated in the ER or in mitochondria (mtUPR), leading to chaperone upregulation and attenuation of protein translation (Braun et al. 2015). In spite of all these proteostatic mechanisms, cell capability to maintain protein homeostasis is compromised with age and chronic stress, resulting in cellular accumulation of protein aggregates (Hipp et al. 2019). It is consensual that these processes contribute to late onset neurodegenerative disorders, like Alzheimer’s disease (AD) (Bruni et al. 2020) and Parkinson’s disease (PD) (Kulkarni et al. 2023), but the specific contribution of protein aggregation to pathological conditions remains a matter of debate and intensive research.

Alzheimer’s disease is the most common form of dementia, affecting over 55 million people worldwide (doubling every 25 years), but research is yet to develop preventive or restorative therapies; as such, in vitro models efficiently mimicking this neuropathology are extremely pertinent for current biomedical research, with obvious societal impact, and will continue to be for the foreseeable future. AD hallmarks include the accumulation of senile plaques and intraneuronal neurofibrillary tangles, resulting from extracellular deposition of the amyloid beta (Aβ) peptide (Glenner & Wong 1984) and intracellular accumulation of hyperphosphorylated tau (Goedert et al. 1992), respectively.

Aβ peptide is a metabolite of the transmembranal Amyloid Precursor Protein (APP) (da Cruz e Silva & da Cruz e Silva, 2003; da Cruz e Silva et al. 2004) that can be sequentially cleaved via alternative pathways (da Cruz e Silva et al. 2004). Cleavage by α-(Gandy et al. 1993) and γ-secretases (Kaether et al. 2006) releases a soluble N-terminal fragment (sAPPα) and a C-terminal fragment (CTF). In this the non-amyloidogenic processing, CTF is subsequently cleaved into P3 and AICD (amyloid precursor protein intracellular domain) (Nhan et al. 2015). In the amyloidogenic processing pathway, APP is alternatively cleaved in the N-terminal domain by β- and γ-secretases, releasing a soluble N-terminal fragment (sAPPβ) and a CTF. This fragment is in turn cleaved, resulting in Aβ40 or Aβ42 peptide (several species are possible outcomes) and AICD (Bukhari et al. 2017; da Cruz e Silva et al. 2004; Seubert et al. 1992; Vitoria et al. 2022; Younkin 1998).

Microtubule-associated tau has 85 phosphorylatable residues, 45 of which have been found to be phosphorylated in the AD brain, notably Thr231, shown to be specific for AD (Oliveira et al. 2017). Thr231 phosphorylation is critical for tau hyperphosphorylation by GSK-3β (Lin et al. 2007): this residue is part of the microtubule-binding tau domain, and its phosphorylation promotes tau-microtubule detachment, decreasing microtubule assembly, with dire functional and homeostatic consequences (Cho & Johnson 2004). As such, evaluation of Thr231 phosphorylation is an important step to identify and characterize ‘in vitro’ AD models.

PD is another significant protein aggregation-related disease, characterized by fibril formation (Lewy Bodies), resulting from aggregation of the protein α-synuclein (Mahul-Mellier et al. 2020). Lewy inclusions are found in regions where cell loss occurs, such as neurons in the substantia nigra, olfactory bulbs, hypothalamus and amygdala nuclei, for example, and are the pathological hallmark of several forms of parkinsonism (Koeglsperger et al. 2023).

Mitochondrial dysfunction is a cellular anomaly that has been associated with several neurodegenerative disorders, including AD and PD (Magalhaes et al. 2021; Murphy et al. 1999; Trigo et al. 2023a, b). The processing and degradation of most proteins is energy dependent, and direct disruption of mitochondria ATP potentiates the accumulation of misfolded proteins, by disturbing the ubiquitin–proteasome system (Solomon & Goldberg 1996), in a process aggravated with ageing (Trigo et al. 2023a, b). In vivo studies in rats have established that treatment with rotenone, an inhibitor of mitochondria complex I, results in dopaminergic degeneration, cytoplasmic α-synuclein and ubiquitin inclusions, and motor deficits, all characteristics of parkinsonian pathogenesis (Alam & Schmidt 2002; Narayanasamy et al. 2004). Moreover, oxidative stress and energy depletion can induce amyloidogenic APP processing, and consequently Aβ peptide production (Busciglio et al. 2002; Sherer et al. 2003), as is the case of H_2_O_2_-induced oxidative stress in human neuroblastoma cells, which results in intracellular Aβ accumulation (Misonou et al. 2000). On the other hand, mitochondrial dysfunctions, observed in AD and PD, can be induced by Aβ peptide (Mossmann et al. 2014) or α-synuclein (Di Maio et al. 2016), in a feedback loop that illustrates the complex relationship between metabolic homeostasis and proteostasis.

In experimental setups, models of protein aggregation are employed to mimic disease states, and thus study the underlying molecular processes associated with pathology. In this study, we characterize four different cellular protein aggregation strategies and compare their responses at a molecular level: protein aggregation was chemically induced by mitochondria complex I inhibition (with the isoflavone rotenone), ATP synthase inhibition (using oligomycin), proteasome inhibition (with MG-132, typically employed to model PD), and treatment with Aβ-peptide (a traditional in vitro model of AD). Although other more complex molecular cell biology models are available, this work focused specifically on four simple models based on cheap and versatile commonly used treatments, frequently used for proof-of-concept and initial studies. The different experimental protocols were evaluated by monitoring the levels of relevant proteins involved in specific aggregation-related diseases.

Moreover, although, the importance of an in vitro AD model based on commercially available cell lines cannot be overstated, a more physiologically significant alternative model would also be of paramount value. One such tool is the use of fibroblasts from human donors, which can be obtained via minimally invasive biopsy, with each cell line reflecting the uniqueness of the donor themself. This work characterises alternative cellular models to mimic AD, one using the neuroblastoma-derived cell line SH-SY5Y, and the other using human fibroblasts from healthy donors.

## Material and Methods

### Antibodies

Primary antibodies used were mouse monoclonal antibody anti-APP, clone 22C11 (1:250, #MAB348, Chemicon International) to detect APP and total sAPP; rabbit polyclonal anti-tau antibody (1:500, #PA5-27,287, Invitrogen) to detect total tau; rabbit monoclonal anti-ptau (Thr231), clone 1H6L6 (1:1000, #701,056, Invitrogen/Life Technologies) to specifically recognize ptau (Thr231) residue; mouse polyclonal anti-α-synuclein antibody (1:400 WB and 1:50 ICC, #sc-12767, Santa Cruz Biotechnology); and rabbit monoclonal anti-HSP-70 antibody (1:1000, #SPA-812, Stressgen Biotechnologies) to detect Heat Shock Protein 70.

Horseradish peroxidase-conjugated anti-mouse (1:5000) or anti-rabbit (1:5000) IgGs were used as secondary antibodies (Amersham Pharmacia) for immunoblotting. Secondary antibodies for immunohistochemistry were anti-mouse and anti-rabbit Alexa Fluor 488 (1:1000; Thermo Fisher Scientific).

### Cell Culture

SH-SY5Y cells (ATCC® CRL-2266™) were grown and maintained in Minimal Essential Medium MEM/F12 1:1 supplemented with 10% Fetal Bovine Serum (FBS), Sodium pyruvate 0.05 g/L and 1% antibiotic–antimycotic. Cultures were maintained in a humidified chamber at 37° C under 5% CO2. Cells were subcultured when 80–90% confluence was reached (Henriques et al. 2009; Santos et al. 2013).

Fibroblasts from 80-years old human donors were commercially obtained (Axol Bioscience) and incubated at 37° C in a humidified atmosphere of 5% CO_2_ and 95% air, cultured with Dulbecco’s Modified Eagle’s Medium (DMEM, Thermo Fisher Scientific) supplemented with 15% fetal bovine serum (FBS, Thermo Fisher Scientific), up to a maximum of 20 passages (Trigo et al. 2023a, b).

Cells were treated between 24 and 72 h after plating, at 70% confluence.

### Treatments

Solutions were prepared in sterile conditions. Aβ1–42 peptide (GenicBio) was dissolved in sterile miliQ water to obtain a 1 mM stock solution and stored at – 20 °C up to a month. Rotenone (Taper) was prepared as a 10 mM stock in DMSO. Oligomycin (Alomone) was dissolved in DMSO to obtain a 5 mM stock solution. MG-132 (Taper) was dissolved in DMSO to obtain a 4 mM stock solution.

Cells were incubated with Aβ1–42 peptide, rotenone, oligomycin and MG-132. Control was incubated with DMSO 0.1%.

Stock solution of Aβ1–42 peptide (1 mM) was aggregated in phosphate-buffered saline (PBS, pH 7.4), for 48 h at 37 °C (final concentration of aggregated stock of 100 µM in 0.9 × PBS) prior to treatment (Henriques et al. 2014; Oliveira et al. 2015). Aggregated peptide was diluted in serum-free medium, before adding directly to the cells in culture, at a concentration of 10 µM, for 24 h. This concentration had already been tested previously in our laboratory (Oliveira et al. 2015).

Stock solutions of rotenone, oligomycin and MG-132 were prepared and diluted in serum-free MEM:F12 (1:1). Rotenone induces a dose-dependent cytotoxic effect, so in this study this mitochondrial inhibitor was added to cells at a concentration of 10 nM for 24 h (Dhanalakshmi et al. 2015). Oligomycin and MG-132 were added to cells at a final concentration of 5 nM and 5 µM, respectively, and left overnight; dose and treatment times were selected in agreement with experience in the laboratory using fibroblasts from human donors (Trigo et al. 2023a, b). Proteasome inhibitor was added to cells at concentrations indicated in Proteostat Aggresome Detection Kit (Enzo Life Sciences).

### Resazurin Assay

For the resazurin assay, SH-SY5Y cells were plated in 96-well plates at a density of 2.0 × 10^4^ cells/well. Following treatments, resazurin dye was added to the cells at a final concentration of 10 µg/mL and incubated at 37° C for 4 h, before reading absorbance intensity at 570 nm using a microplate reader.

### Sample Collection and Protein Analysis

Cells were plated in 6-well plates at a density of 8.0 × 10^5^ cells/well. Following treatments, cells were washed with ice-cold PBS and collected in RIPA lysis buffer (Sigma) containing a protease inhibitor cocktail (cOmplete, EDTA-free, Roche) and supplemented with NaF (7.5 mM) and Na_3_VO_4_ (1 mM). Cell lysates were sonicated (two cycles of 5 s) and stored at −20° C. A BCA assay kit (Alfagene) was used to determine protein concentration and samples were normalized for protein content. Conditioned media was collected into Sodium Dodecyl Sulfate (SDS) (final concentration 1%) and incubated at 100 ⁰C for 10 min.

Samples were electrophoretically separated using 5–20% gradient SDS-PAGE gels and transferred to a nitrocellulose membrane, followed by immunoblotting for proteins of interest. To summarize, 50 µg total protein were loaded per well per sample and further normalized to ponceau. Detection was performed using the chemiluminescent method with ECL (Amersham pharmacia), and bands were quantified with Chemidoc Touching Images (Bio-Rad). Protein bands were quantified using ImageLab 6.0.1 software.

### Immunofluorescence Microscopy

Cells were cultured on coverslips in 12-well plates (1.8 × 10^5^ cells/well), and washed twice with PBS, after treatment, and fixed with 4% formaldehyde for 20 min. Cells were subsequently washed and permeabilized with 0.5% triton X-100 and EDTA 3 mM (pH 8) for 30 min with shaking at 4 ⁰C. After permeabilization, cells were washed with PBS and stained with Proteostat Aggresome Detection Kit (1:3000) (Enzo Life Sciences) for 30 min at room temperature, to detect aggregated proteins. Following sample mounting, with Vectashield Mounting Medium with DAPI (Vector Labs), cells were imaged by fluorescence microscopy using Zeiss Axioimager Z1 microscope. Four fields were randomly analyzed from 3 independent experiments for each condition, samples were scored for protein aggregation positive cells.

For immunocytochemistry, fixed cells were incubated with ptau (Thr231) (1:500) or α-synuclein (1:50) antibodies for 3 h prior to proteostat staining, following which, preparations were incubated with Alexa Fluor 488 (Invitrogen) secondary antibody before mounting, and imaging was carried out as previously described (Santos et al. 2013).

In some preparations, images were captured using a Zeiss LSM 880 laser‐scanning confocal microscope (Carl Zeiss, Oberkochen, Germany) with a × 63 oil‐immersion Aprochromat objective with an image size of 512 × 512 pixels, with a pinhole aperture of 1 Airy unit. Settings for gain, contrast, and brightness were optimized initially and held constant throughout each study so that all sections were digitized under the same conditions. Z-stacks were set so that the whole cell was imaged, in 1 µm intervals.

### Quantification and Statistical Analysis

Image Lab 6.0.1 software (Bio-Rad) was used to quantify band intensity in immunoblots and correlate it with protein levels. Data are expressed as mean ± SEM determinations, from at least three independent experiments. Statistical analysis was carried out by one-way ANOVA. When F values were significant, Dunnett test was applied to compare all groups vs. control. For Aβ experiments in human fibroblasts, one-sample t-test analysis was performed. Colocalization studies were performed by the Pearson product-moment correlation coefficient (Pearson's r value), obtained from the Coloc 2 plugin for ImageJ-Fiji. The level of significance accepted was *P < 0.05; **P < 0.01 and ***P < 0.001.

## Results

### Mitochondrial Disruption Fails to Increase Overall Protein Aggregation in a Neural Cell Line

Several different in vitro models are currently being explored to model protein aggregation-associated neuropathologies, but no thorough comparison between these has yet been described. In this work, we explore the four most common types of aggregation protocols: pathology-associated mechanism (treatment with aggregated Aβ1–42 peptide), redox imbalance (inhibition of mitochondrial respiratory chain complex I with rotenone), energy dysfunction (inhibition of mitochondrial ATP synthesis with oligomycin), and direct proteostasis disruption (proteosome inhibition with MG-132).

**Fig. 1:**
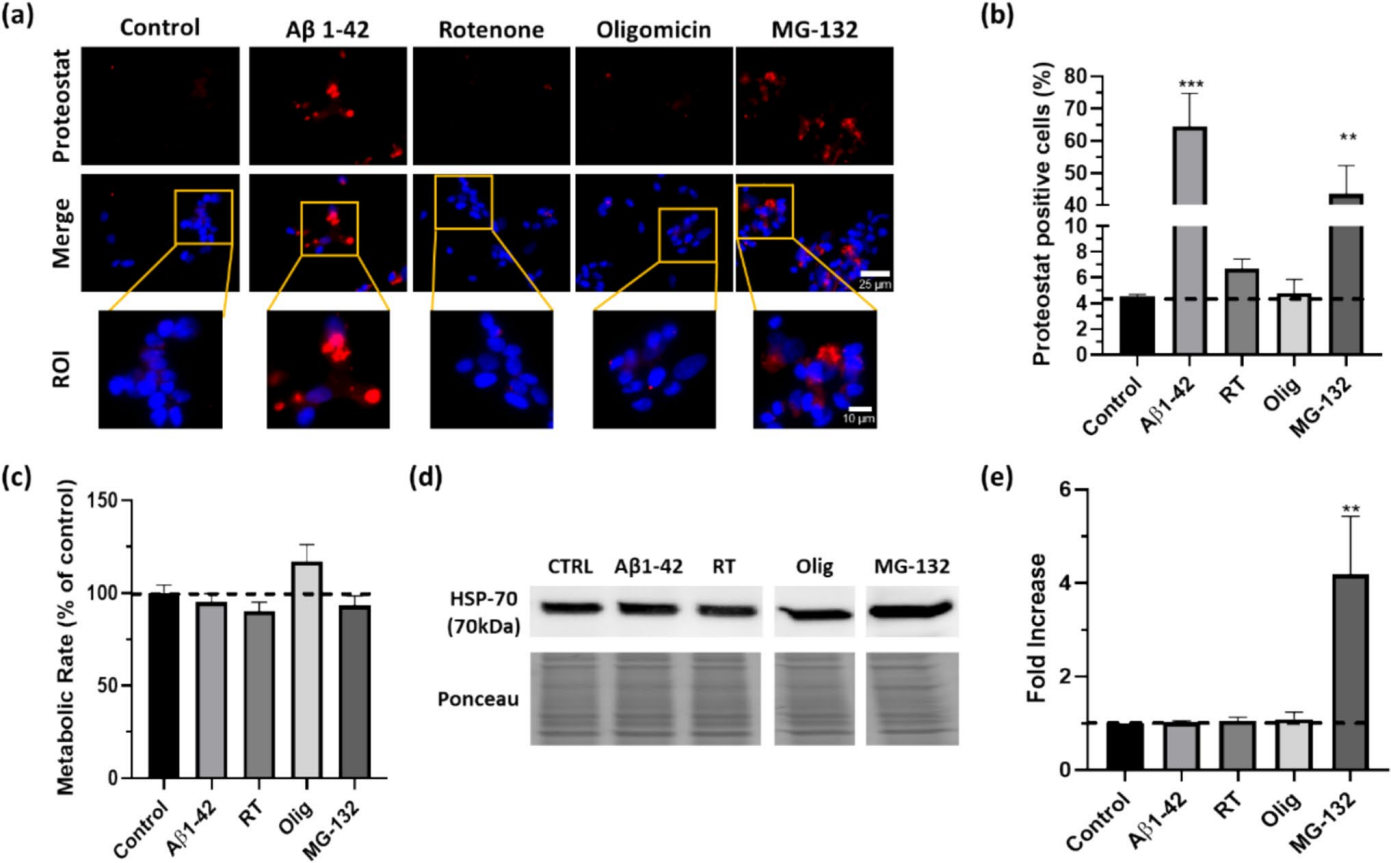
Comparative proteasome inhibition induced protein aggregation and alterations in HSP-70 levels in SH-SY5Y cells. SH-SY5Y cells were exposed to 10 µM of Aβ1–42 peptide (priorly aggregated for 48 h at 37° C), 10 nM of rotenone (electron transport chain inhibitor, RT) for 24 h, 0.5 µM of oligomycin (ATP synthase inhibitor, Olig) or 5 µM of MG-132 (Proteasome inhibitor) overnight. Aggregation was monitored by immunofluorescence microscopy using a proteostat dye (**a**). Fluorescence images were acquired with Zeiss microscope. Representative examples are shown for each experimental condition. Scale bar is 25 µm in top and 10 µm in bottom panels. Four fields were randomly acquired for each condition and proteostat positive cells were counted (**b**). Metabolic rate was measured using the resazurin solution after cells were exposed to treatments and expressed as a function of control, where control was a set at 100% (**c**). HSP-70 was detected by subjecting the cellular lysates to western blot analysis with specific antibody (**d**). Quantitative results are expressed as mean ± SEM of three independent experiences. ***P < 0.001, significantly different from control; *P < 0.05, significantly different from control. Dunnett post hoc test (**e**)

Cells from the human neuroblastoma cell line SH-SY5Y were thus treated with aggregated Aβ1–42 peptide, rotenone, oligomycin and MG-132. The first parameter evaluated was overall protein aggregate formation. Protein aggregation was measured by fluorescence microscopy, using the proteostat aggresome detection dye. To assist colocalization, nuclei were stained with DAPI (Fig. 1a).

The most marked response was obtained upon incubation with aggregated Aβ1–42 peptide, which resulted in a dramatic 14-fold increase in protein aggregation, when compared to control cells, with large aggregates being detected in most observed cells (Fig. 1a and b). These aggregates seem related to specific cellular processes, as acute treatment with oligomerised Aβ1–42 (5 min) failed to elicit any alterations in proteostat signalling (supplementary Fig. 1).

Mitochondrial function and ATP production are essential for protein degradation, and mitochondria dysfunction is an important mechanism underlying several neuropathologies. Thus, to evaluate the effects of oxidative stress, mitochondrial dysfunction, and ATP depletion in protein aggregation, SH-SY5Y were treated with rotenone and oligomycin. Rotenone is an inhibitor of mitochondrial complex I, promoting electron accumulation in the mitochondrial matrix and resulting in increased reactive oxygen species (ROS) production (Heinz et al. 2017). Oligomycin induces ATP depletion by inhibiting ATP synthase activity. However, despite the above-described effects on mitochondrial dysfunction, no significant differences were observed in aggregate formation upon cell treatment (although rotenone appeared to reveal a small increase) (Fig. 1a and b). The fact that both treatments failed to promote protein aggregation in SH-SY5Y cells is a surprising result, considering that these treatments are often used in cellular models to study the molecular basis of aggregate forming disorders (Chaves et al. 2010; Lee et al. 2002).

The final aggregation model tested relies on the proteasome inhibitor MG-132. Due to its mode of action, this aldehyde did elicit a strong response in SH-SY5Y cells. Incubation with this compound resulted in a dramatic tenfold increase in protein aggregation (Fig. 1a and b).

Resazurin assay, commonly used to assess the number of live cells in a sample and monitor cell viability / cytotoxicity, indicated that cells were not particularly affected in terms of viability with any treatments (Fig. 1c), with oligomycin being the only condition to show a small, not statistically significant (*p* = 0.1351), increase in metabolic rate, compared to control conditions.

Protein folding/misfolding and aggregation can be regulated by different mechanisms; in cells under proteotoxic stress, the activation of the UPR is a significant process, as is consequent upregulation of chaperones. The UPR-associated chaperone HSP-70 is expressed in response to stress; its levels in the different models were thus assessed by western-blotting.

A larger than fourfold increase was observed with proteasome inhibition, by MG-132, reiterating the dysregulation of protein homeostasis and activation of UPR (Fig. 1d and e). In contrast, treatments with Aβ, rotenone, or oligomycin did not alter the levels of HSP-70 (Fig. 1d and e), clearly highlighting that the underlying mechanisms of aggregate formation by Aβ and MG-132 are distinct.

### Aggregate Heterogeneity: Different Conditions Model Different Physiological Phenomena

Having determined that aggregates formed by Aβ and MG-132 appear to arise by distinct processes, deposit morphology was then analysed. Aggregates are morphologically distinct, with Aβ-induced aggregates being larger and tending to agglomerate as a single deposit (Fig. 1a), reinforcing the relevance of this model to study AD related events, reminiscent of processes associated with fragment accumulation (Bukhari et al. 2017; Younkin 1998). In contrast, MG-132 induces several smaller aggregate punctate formations, consistent with processes associated with the UPR response and oxidative stress. As already described, oligomycin and rotenone produce only a few aggregates, and when formed these resemble those obtained with the latter.

**Fig. 2:**
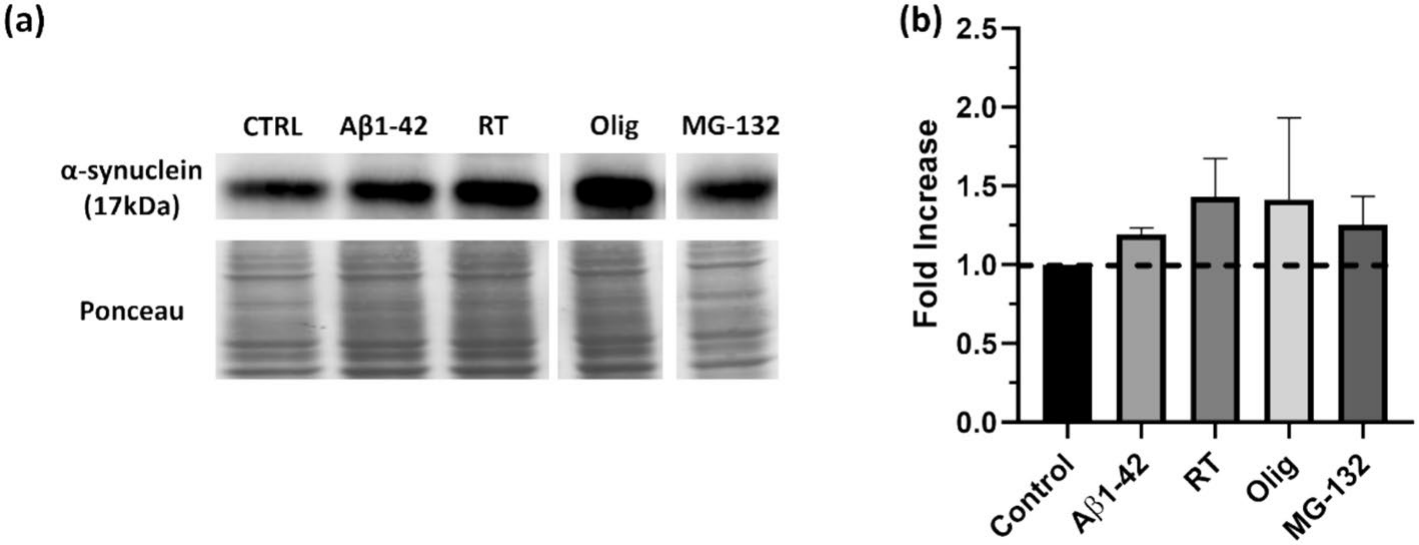
Aggregated Aβ1–42 peptide, mitochondrial dysfunction and proteasomal inhibition impact α-synuclein levels in SH-SY5Y cells. α-Synuclein levels were evaluated in SH-SY5Y cells by western blot (**a**) and quantified (**b**) Aβ1–42 was aggregated for 48 h at 37° C. Cells were treated with Aβ1–42, RT, Olig and MG-132. α-synuclein was detected by subjecting the cellular lysates to western blot analysis (**a**) with specific antibody. Quantitative results are expressed as mean ± SEM of three independent experiments (**b**)

Disease-specific protein aggregates are a histopathological hallmark of several neuropathologies, distinguished by the type of deposited protein. PD is notably characterised by the formation of Lewy bodies, resulting from α-synuclein aggregation (Koeglsperger et al. 2023); being so, α-synuclein levels were evaluated by western blot analysis to characterize these aggregation protocols as possible PD models (Fig. 2). α-synuclein levels seemingly tend to increase with all aggregation models tested, as determined by western blot analysis; strikingly, the treatments appearing to provoke the highest response in terms of aggregation formation (Aβ and MG-132) were not the ones promoting more α-synuclein accumulation.

Mitochondria dysregulation is tightly associated with neurodegeneration in PD (Magalhaes et al. 2021; Pozo Devoto & Falzone 2017) and some mitochondria-damaging pesticides have been linked with PD incidence and parkinsonian-symptoms (Elbaz & Moisan 2008), including α-synuclein deposition and aggregation (Nistico et al. 2011). As such, results obtained with rotenone treatment, the most effective protocol to induce the formation of α-synuclein aggregates (Fig. 2 and supplementary Fig. 2), are consistent with the current state-of-the-art and reinforce the notion that rotenone is in fact a good cellular model for PD.

### Aggregate Formation is Associated with Increases in tau Thr231 Phosphorylation in SH-SY5Y Cells

**Fig. 3:**
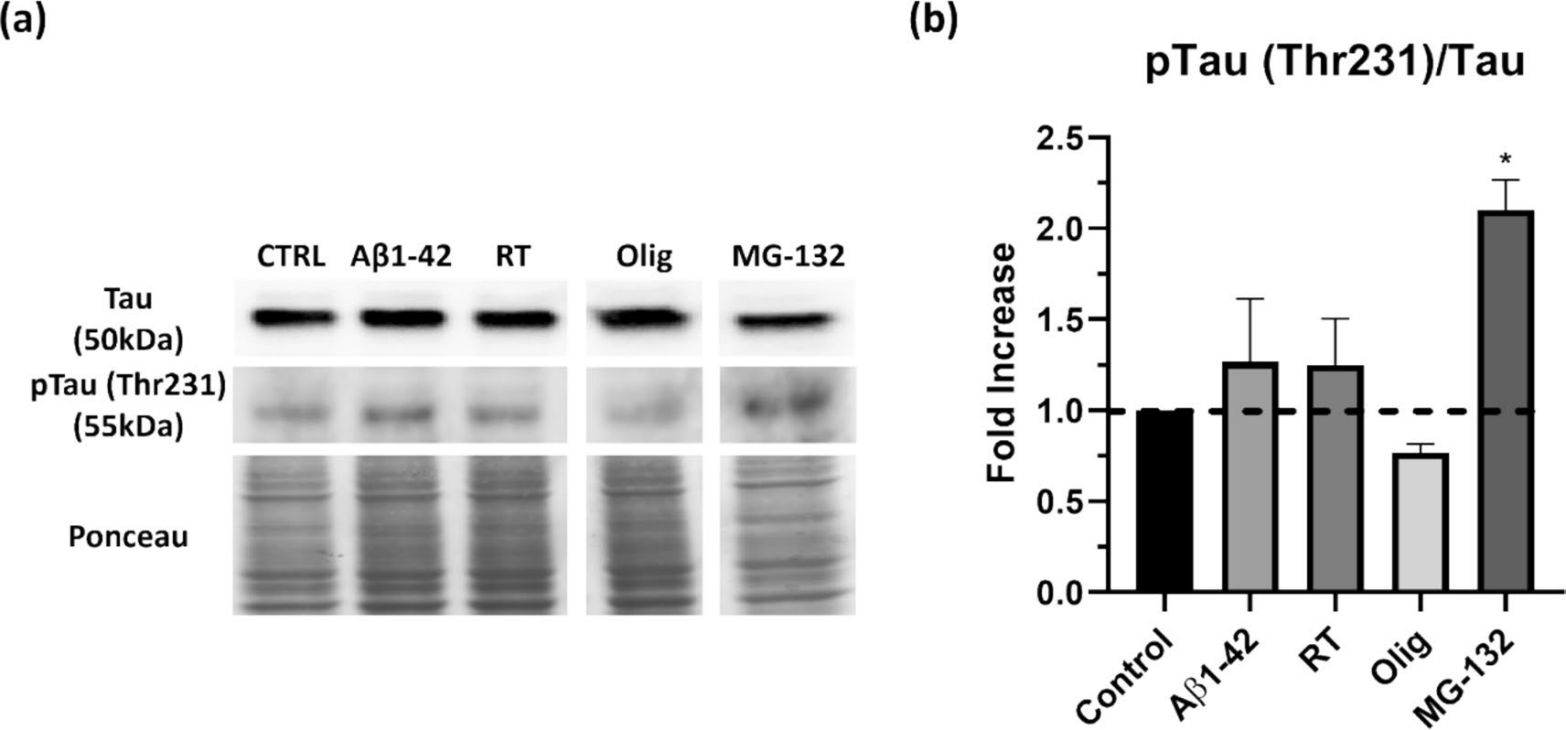
Proteasomal inhibition induces an increase in pTau (Thr231)/TAU in SH-SY5Y cells. pTau (Thr231) and Tau protein levels were evaluated in SH-SY5Y cells by western blot. Cells were treated with aggregated Aβ1–42, RT, Olig and MG-132. pTau (Thr231) and Tau were detected by subjecting the cellular lysates to western blot analysis with specific antibodies (**a**). pTau (Thr231)/Tau ratio was also quantified (**b**). Quantitative results are expressed as mean ± SEM of four independent experiences. *P < 0.05, significantly different from control. Dunnett post hoc test

The formation of protein aggregates is often associated with protein phosphorylation- and hyperphosphorylation-related events. Considering the results of mitochondrial dysfunction and proteasomal inhibition, and given the association between AD and tau phosphorylation, its levels were then evaluated. Results revealed that proteasome inhibition with MG-132 had the greatest increase in tau phosphorylation (Fig. 3a), with an almost twofold increase in ptau/tau ratio (*p* = 0.0187) (Fig. 3b).

Considering that tau phosphorylation diminishes its microtubule-binding ability and leads to aggregation, the increased ptau/tau ratio resulting from MG-132 treatment can ultimately be resulting in neurofibrillary tangle formation. Although not statistically significant, there appears to be a tendency for Aβ to increase tau phosphorylation; our group had previously described tau phosphorylation to increase in neuronal primary cultures treated with aggregated Aβ1–42 (Oliveira et al. 2015), and immunofluorescence studies suggest phosphorylated tau to localize to the cellular aggregates formed after Aβ exposure (Supplementary Fig. 1). However, the physiological significance of increased tau phosphorylation in this context remains unclear, as there does not appear to be accompanied by increased total tau accumulation in aggresomes (supplementary Fig. 3). On the other hand, aggresomes characteristically accumulate as consequences of proteasome inhibition (Trigo et al. 2023a, b); as such, it is not particularly surprising that investigation for tau colocalization with aggresomes revealed a decrease in cells treated with proteasome inhibitor MG132 (supplementary Fig. 3c).

Pathological tau phosphorylation is intimately associated with mitochondria dysfunction. Hyperphosphorylated and aggregated tau disrupts mitochondrial axonal transport and dynamics, while mitochondrial dysfunction promotes tau pathology in AD (Cheng & Bai 2018). Our results revealed a marginal increase in tau phosphorylation with rotenone, albeit not statistically significant (*p* = 0.8334); however, treatment with oligomycin appears to decrease the ratio of ptau (Thr231) phosphorylation (Fig. 3b).

### APP Metabolism can be Modulated not only by Aβ1–42, but also by Proteosome Inhibition

**Fig. 4:**
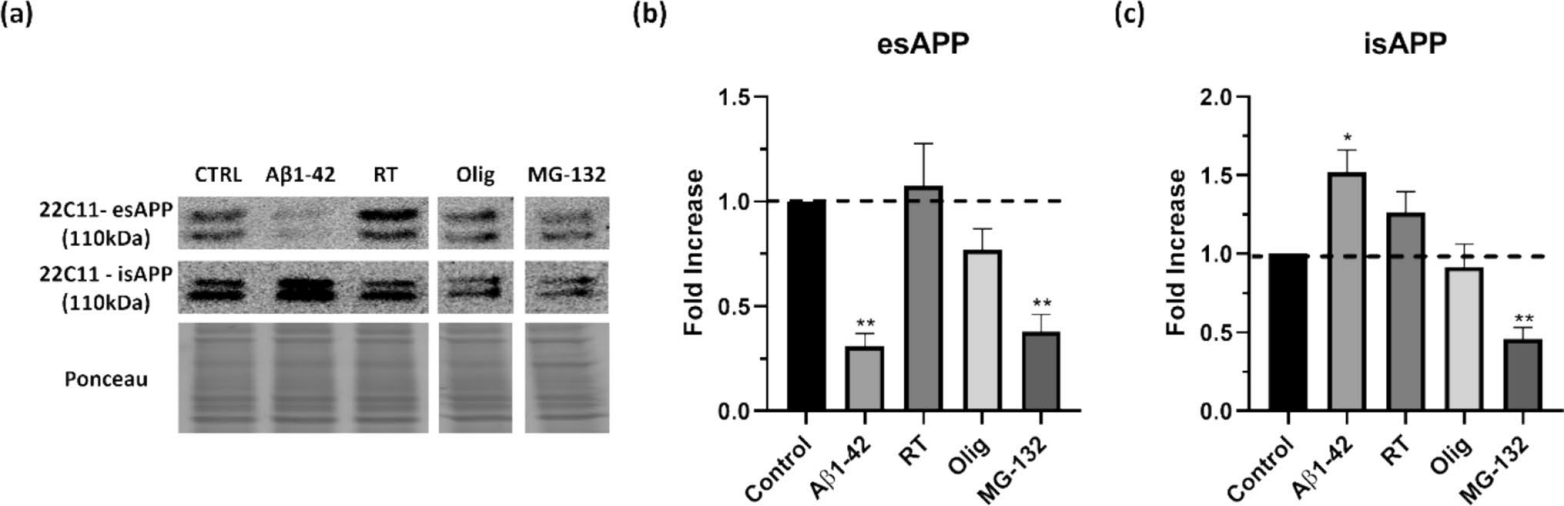
Aggregated Aβ1–42 induces anomalies in APP metabolism. Extracellular APP (esAPP) and intracellular APP (isAPP) levels were evaluated in SH-SY5Y cells. Cells were treated with aggregated Aβ1–42, RT, Olig and MG-132 and esAPP and isAPP were detected by subjecting the conditioned cell medium (a, quantified in b) or cellular lysates (a, quantified in c), respectively, to western blot analysis with 22C11 antibody. Quantitative results are expressed as mean ± SEM of at least three independent experiences. *P < 0.05, **P < 0.01 significantly different from control. Dunnett post hoc test

Tau, one of the key proteins involved in AD, is clearly directly affected by the aggregation protocols here assessed. The other protein more obviously linked to AD is APP; as such, procedures were set up to evaluate if proteasomal inhibition and mitochondrial dysfunction can alter APP levels in SH-SY5Y cells. The APP monoclonal antibody 22C11 was used in conditioned medium to assess extracellular secreted APP (esAPP), and cell lysates to detect holo- and intracellular secreted APP (hAPP and isAPP, respectively) (Fig. 4a).

Proteasomal inhibition induced by MG-132 modulates both the levels of esAPP and isAPP (Fig. 4b and c, respectively), while oligomycin appears to perhaps marginally decrease APP secretion (measured by assessing APP presence in conditioned media), with no variations in intracellular APP levels (Fig. 4c). No alterations in esAPP were observed with rotenone (Fig. 4b), but isAPP appears to be slightly increased (Fig. 4c). MG-132 provoked a more marked decrease in secreted APP and in intracellular APP levels, suggesting that it is probably affecting APP transcription itself.

Fitting with our own previous work, the most marked effect was observed with the AD model of treatment with Aβ1–42 peptide, which resulted in a decrease in esAPP (Fig. 4b), contrasted by an increase in intracellular APP (Fig. 4c). We have previously described this accumulation of intracellular APP to result from APP not being secreted (Henriques et al. 2009), and the consistent results here presented suggest that proteasomal inhibition and mitochondrial dysfunction can be important to study other protein-aggregation-related disease.

**Fig. 5:**
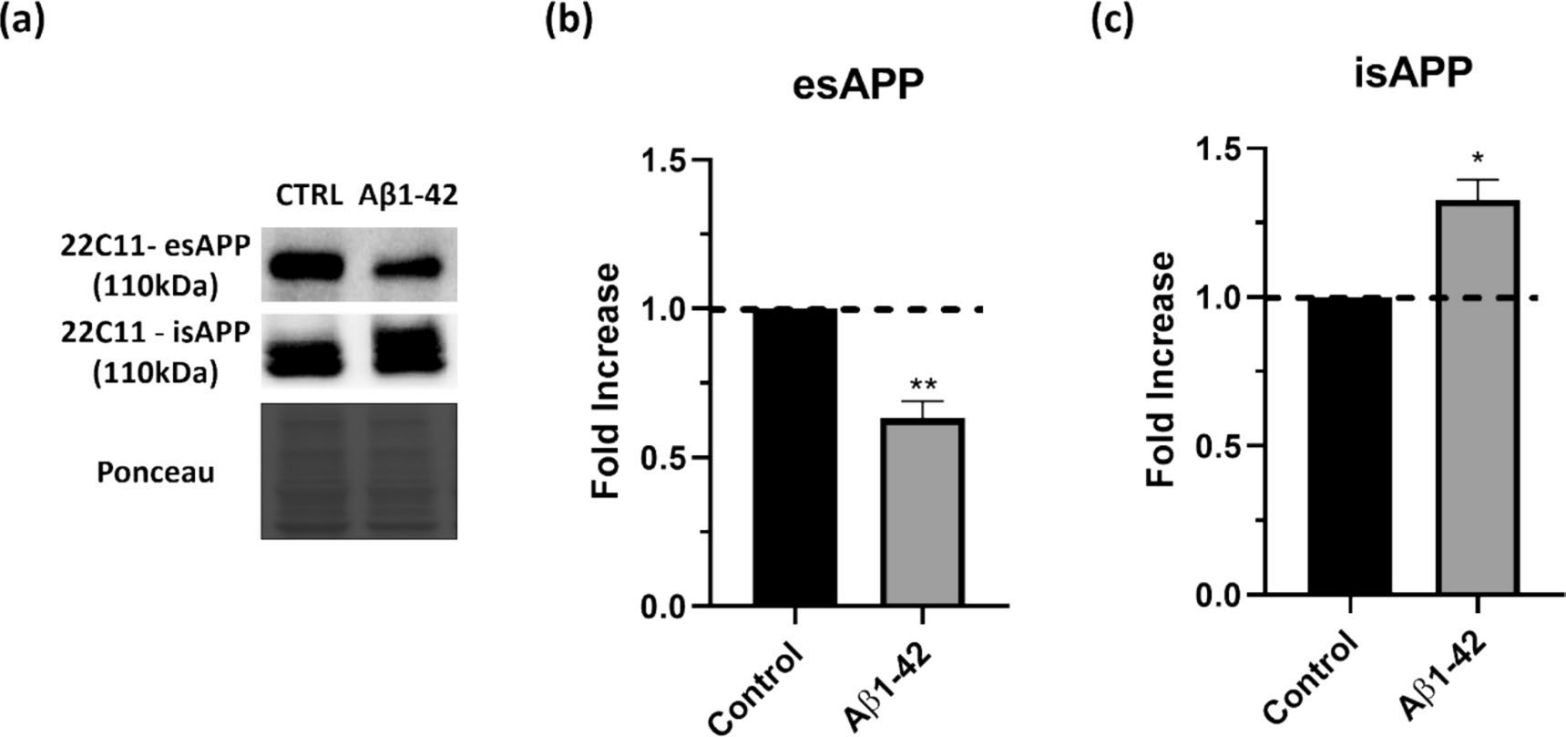
Aggregated Aβ1–42 induces anomalies in APP metabolism in Human Fibroblasts. Extracellular APP (esAPP) and intracellular APP (isAPP) levels were evaluated in Human fibroblast. Cells were treated with aggregated Aβ1–42 and esAPP and isAPP were detected by subjecting the conditioned cell medium (a, quantified in b) or cellular lysates (a, quantified in c), respectively, to western blot analysis with 22C11 antibody. Quantitative results are expressed as mean ± SEM of at least three independent experiences. *P < 0.05, **P < 0.01 significantly different from control. One-sample t- test

Considering the AD-relevance of this aggregation model based on a cell line, we investigated whether a similar result could be obtained using human cells. Indeed, treatment of fibroblasts from an 80-year-old human healthy donor with aggregated Aβ1–42, mirrored the results obtained in SH-SY5Y cells (Fig. 5), with a significant decrease in extracellular APP (Fig. 5b) and an increase in intracellular APP (Fig. 5c).

## Discussion

The focus of this work was the characterisation of different cell models commonly used to study protein aggregation and neurodegenerative disorders.

Incubation of the neuroblastoma-derived cell line SH-SY5Y with aggregated Aβ1–42 for 24 h greatly increases protein aggregation, relatively to control cells, even more so than proteasome inhibition (Fig. 1a), confirming it as a good model to study the protesostasis-related aspects in AD. Acute treatment with aggregated Aβ1–42 failed to affect protein aggregation, indicating that oligomerised Aβ1–42 must be processed intracellularly to induce aggregation.

The different models feature distinct aggregates (Fig. 1a). Those induced by Aβ are typical of aggregates formed by the accumulation of peptides: this is the case of Aβ aggregation, but a similar process occurs with the accumulation of huntingtin fragments (Bauer et al. 2012; Cyr et al. 2006) or synphilin-1 (Ferreira-Fernandes et al. 2015). The fact that the larger aggregates were obtained upon Aβ addition is consistent with literature, as it has been well reported that aggregated Aβ recruits further fragments to the deposits (Itoh et al. 2022) possibly even including secreted APP (Henriques et al. 2009). Given the described aggregate characteristics, it is reasonable to posit that Aβ approximates aggregate formation associated with AD.

The chaperone HSP-70 plays crucial roles in proteostasis, and is currently being explored as a good target to modulate the AD-phenotype, with studies suggesting shown that AD pathology can be supressed by increasing cellular levels of HSP-70 (Martin-Pena et al. 2018; Morimoto & Cuervo 2014; Venediktov et al. 2023). Our results show no differences in HSP-70 levels after treatment with the Aβ peptide, contrary to what was observed with MG-132, indicating that Aβ and MG-132 trigger distinct aggregate formation mechanisms.

We have previously described this model to increase tau phosphorylation in rat primary cortical neuronal cultures. Tau has 85 phosphorylatable residues (45 of which found in AD brains) (Oliveira et al. 2017); however, it has been demonstrated that residue Thr231 is the primary phosphorylation site for GSK-3β and other kinases, such as cdk5, critical for tau hyperphosphorylation, and phosphorylation at this residue can destabilize tau-binding to microtubules and promote tau aggregation (Lin et al. 2007) The effect of Aβ in modulating protein kinases such as GSK-3β and cdk5 is well established, and these kinases are believed to directly contribute to tau hyperphosphorylation (Oliveira et al. 2015). Although staining for total tau protein indicates that tau presence in aggresome remains unaltered (supplementary Fig. 3), and in only a slight increase in Thr231 phosphorylation was suggested in response to incubation with oligomerised Aβ1–42, the phosphorylated form appeared to colocalize intensely with increased aggresomes, suggesting that phospho-tau (a fraction of all tau protein in the cell) is aggregating or being sequestrated in protein aggregates (Supplementary Fig. 4). Perhaps one could anticipate increased tau phosphorylation to result in increased tau colocalization with aggresome, but results indicate otherwise. A possible explanation is that oligomerised Aβ1–42 is greatly increasing protein aggregation as a whole, masking the small observed increase in tau Thr 231 phosphorylation, which would represent a small fraction of aggregated proteins.

The Aβ1–42 peptide model also exhibits a decrease in extracellular APP and increase in intracellular APP, in both neural (Fig. 4) and non-neural (Fig. 5) cells lines, suggesting that APP metabolism is hindered, accordant to our previous work with primary neuronal cultures (Henriques et al. 2014).

High levels of α-synuclein in cerebrospinal fluid (CSF) have been reported in patients with AD and mild cognitive impairment. Consistently, our results suggest an increase in α-synuclein levels (Fig. 2) and aggregation (Supplementary Fig. 2) upon treatment with Aβ1–42 peptide, strengthening the relevance of this model to study different protein aggregation-related phenotypes.

With aging, mitochondrial function is compromised, impairing metabolism and increasing oxidative stress (Trigo et al. 2019). Mitochondria-produced ATP is important for several processes of protein degradation and homeostasis; being so, manipulation of mitochondria is a sensible strategy to develop protein-aggregation cell models (Clare & Saibil 2013; Solomon & Goldberg 1996). Rotenone was used to induce oxidative stress in SH-SY5Y by inhibiting the mitochondrial electron chain transport, promoting electron accumulation and consequently increasing ROS levels (Sherer et al. 2003). Using a parallel approach, ATP production was disrupted by inhibiting ATP synthase with oligomycin (Jastroch et al. 2010). Although no significant differences were observed in protein aggregation with both chemicals, a small increase in aggregation occurs with rotenone, which induced smaller aggregate punctate formation, like MG-132 (Fig. 1). Recent studies in our laboratory described that rotenone-induced protein aggregation in human fibroblasts can be rescued by caloric restriction (Trigo et al. 2023a, b), and previous work has linked starvation with improved mitochondria activity, reducing oxidative stress (Yun et al. 2020), which may also contribute to the observed results. Moreover, our results suggest that mitochondria manipulation induces protein aggregation through mechanisms distinct from aggregated Aβ1–42, as it fails to fully induce the AD-phenotype observed with that model.

Another relevant point is that rotenone-induced mitochondrial dysfunction can potentiate accumulation of misfolded proteins, like α-synuclein, being a commonly used model of PD (Alam & Schmidt 2002; Narayanasamy et al. 2004; Sherer et al. 2003), and our results suggest this pesticide might be increasing the levels and aggregation of α-synuclein. While the physiological action of α-synuclein is important for mitochondrial homeostasis, its pathological aggregation can negatively impinge on mitochondrial function (Faustini et al. 2017, 2019): soluble α-synuclein is targeted to mitochondria (Guardia-Laguarta et al. 2014), and the interaction of oligomeric α-synuclein with mitochondria results in decreased respiration (Nakamura et al. 2011). Taken together, results support the use of rotenone as a good model to study PD.

The molecular chaperone HSP-70 inhibits α-synuclein fibril formation and alters the characteristics of toxic α-synuclein aggregates (Dedmon et al. 2005), with studies in animal models describing rotenone exposure to decrease HSP-70 expression (Sonia Angeline et al. 2012); in various diseases, aggregation-induced toxicity is supressed by HSP-70 overexpression, reinforcing its important role in aggregation-related disease (Kundel et al. 2018; Meriin et al. 2018). However, no differences in HSP-70 levels were observed in SH-SY5Y cells after rotenone exposure, suggesting that other mechanisms of UPR can be altered and be responsible for the observed increase in α-synuclein aggregation.

Cellular homeostasis is maintained via ubiquitin–proteasome and lysosomal-autophagic systems to prevent protein aggregation (Trigo et al. 2019). The aldehyde MG-132 binds to the active site of the proteasome, inhibiting its activity (Guo & Peng 2013), and is frequently used as a positive control for protein aggregation(Bang et al. 2014). As expected, protein aggregation increases with proteasome inhibition (Fig. 1), an increase that is accompanied by an increase in tau phosphorylation in residue Thr231. Tau hyperphosphorylation is a histological hallmark of many neurodegenerative disorders that can ultimately form tau aggregates, and proteasome inhibition has been reported to promote degradation and oligomeric accumulation of tau (Hamano et al. 2009). Aggregates produced by MG-132 are typical of aggregates containing ubiquitinated proteins, known as aggresome-like induced structures (ALIS) (Vasconcellos et al. 2016). These results are consistent with the state-of-the art, given the effect of MG-132 as a proteasome inhibitor.

On the other hand, work using neuroblastoma cell lines reports proteasome inhibition to result in tau proteolysis (Delobel et al. 2005), also consistent with the decrease in the levels of total tau observed in this work (Fig. 3). In parallel to decreased tau levels, APP levels (both intra- and extracellular) are also decreased by MG-132 (Fig. 4). A cellular safety mechanism to cope with increased protein aggregation is to reduce protein transduction, which can explain these results. Moreover, proteasome inhibition has been described to promote a reduction in mature APP/APLP1 (amyloid beta precursor-like protein 1) via autophagy induction (Zhou et al. 2011).

In contrast to the other models explored in this study, HSP-70 expression was found to increase with proteasome inhibition, in agreement with other studies reporting MG-132 to result in accumulation of ubiquitinated proteins and increase in HSPs expression (Young & Heikkila 2010).

To conclude, this study establishes and characterizes different cellular models for protein aggregation-related diseases, which we believe are of great value for studies in related research fields. By adequately applying these protocols to model the more appropriate disease or biological process, several additional scientific questions and disease-related mechanisms can be explored.

## Supplementary Information

Below is the link to the electronic supplementary material.

Supplementary file1 (PPTX 57607 KB)

## Data Availability

Data are available from the corresponding author on reasonable request
