# Supplementary material for: Comparing In vitro Protein Aggregation Modelling Using Strategies Relevant to Neuropathologies
Source: Cell Mol Neurobiol. 2025 Mar 13;45:24. doi: 10.1007/s10571-025-01539-z (PMC11906958; doi:10.1007/s10571-025-01539-z)
Supplement: Supplementary file 1 — Supplementary file1 (PPTX 57607 KB) [file 10571_2025_1539_MOESM1_ESM.pptx]

## Slide 1
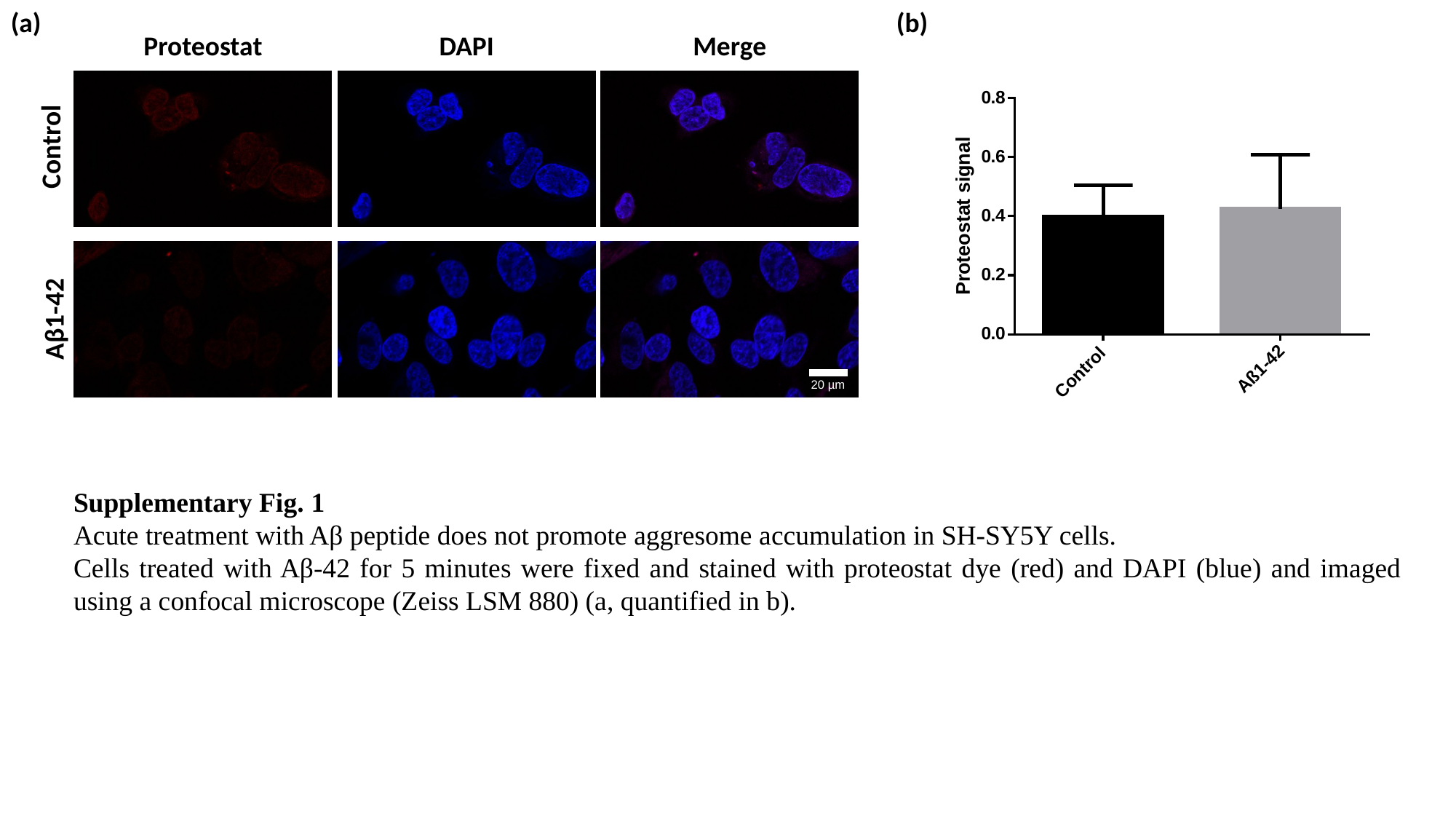

(a)
(b)
Proteostat
DAPI
Merge
Control
Aβ1-42
20 µm
Supplementary Fig. 1
Acute treatment with Aβ peptide does not promote aggresome accumulation in SH-SY5Y cells.
Cells treated with Aβ-42 for 5 minutes were fixed and stained with proteostat dye (red) and DAPI (blue) and imaged using a confocal microscope (Zeiss LSM 880) (a, quantified in b).

## Slide 2
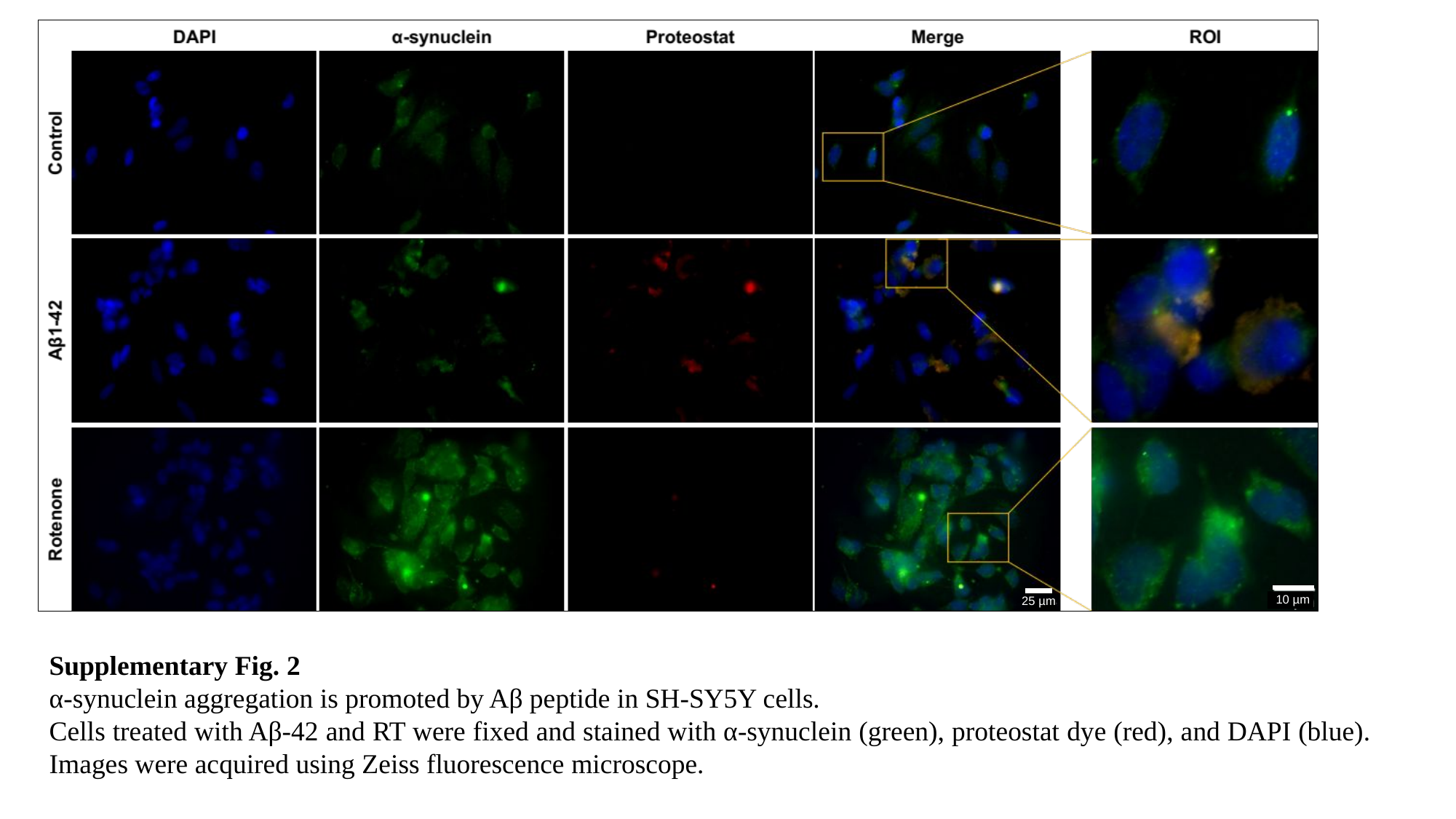

10 µm
25 µm
Supplementary Fig. 2
α-synuclein aggregation is promoted by Aβ peptide in SH-SY5Y cells.
Cells treated with Aβ-42 and RT were fixed and stained with α-synuclein (green), proteostat dye (red), and DAPI (blue). Images were acquired using Zeiss fluorescence microscope.

## Slide 3
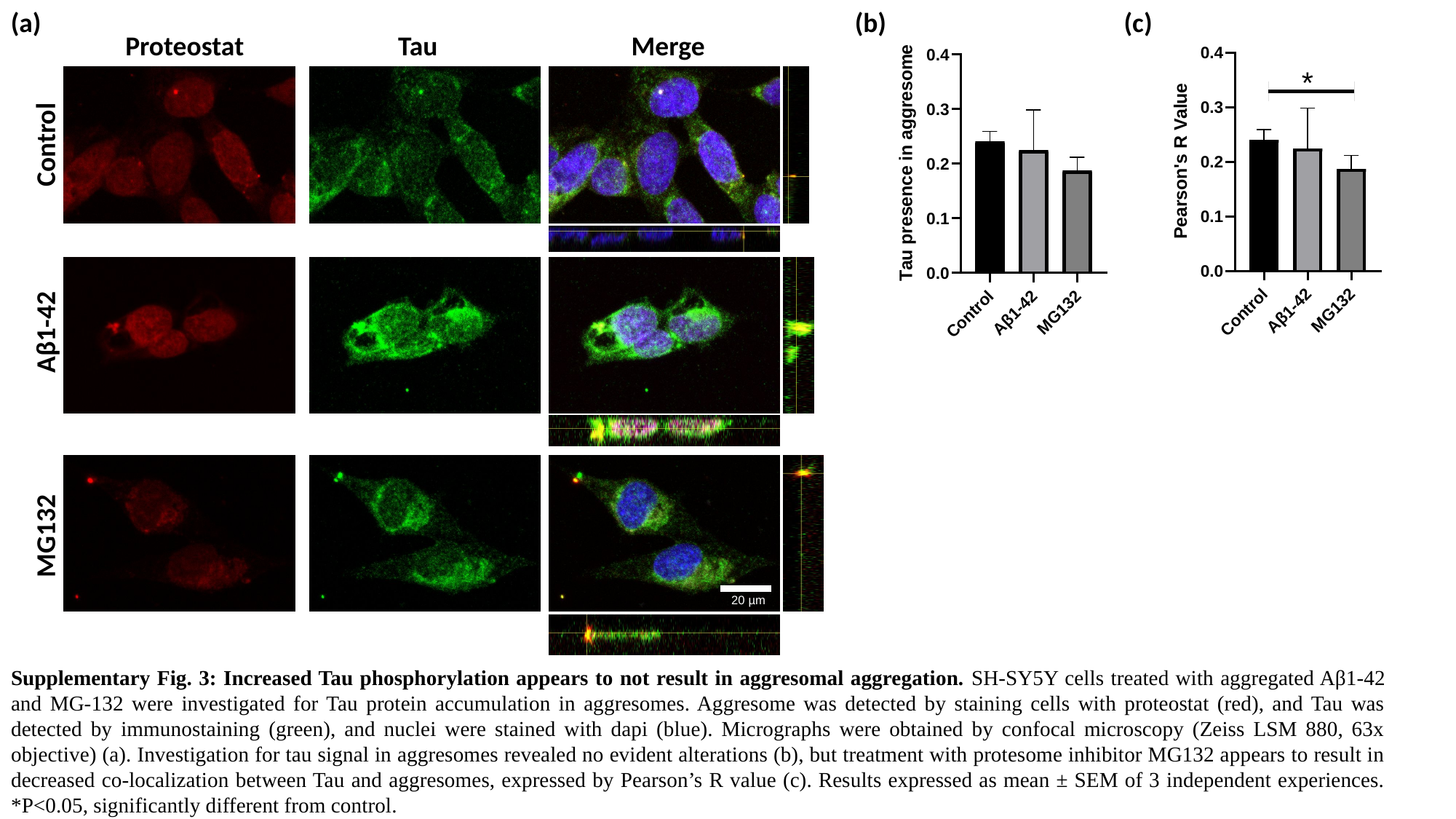

(a)
(b)
(c)
Proteostat
Tau
Merge
Control
Aβ1-42
MG132
20 µm
Supplementary Fig. 3: Increased Tau phosphorylation appears to not result in aggresomal aggregation. SH-SY5Y cells treated with aggregated Aβ1-42 and MG-132 were investigated for Tau protein accumulation in aggresomes. Aggresome was detected by staining cells with proteostat (red), and Tau was detected by immunostaining (green), and nuclei were stained with dapi (blue). Micrographs were obtained by confocal microscopy (Zeiss LSM 880, 63x objective) (a). Investigation for tau signal in aggresomes revealed no evident alterations (b), but treatment with protesome inhibitor MG132 appears to result in decreased co-localization between Tau and aggresomes, expressed by Pearson’s R value (c). Results expressed as mean ± SEM of 3 independent experiences. *P<0.05, significantly different from control.

## Slide 4
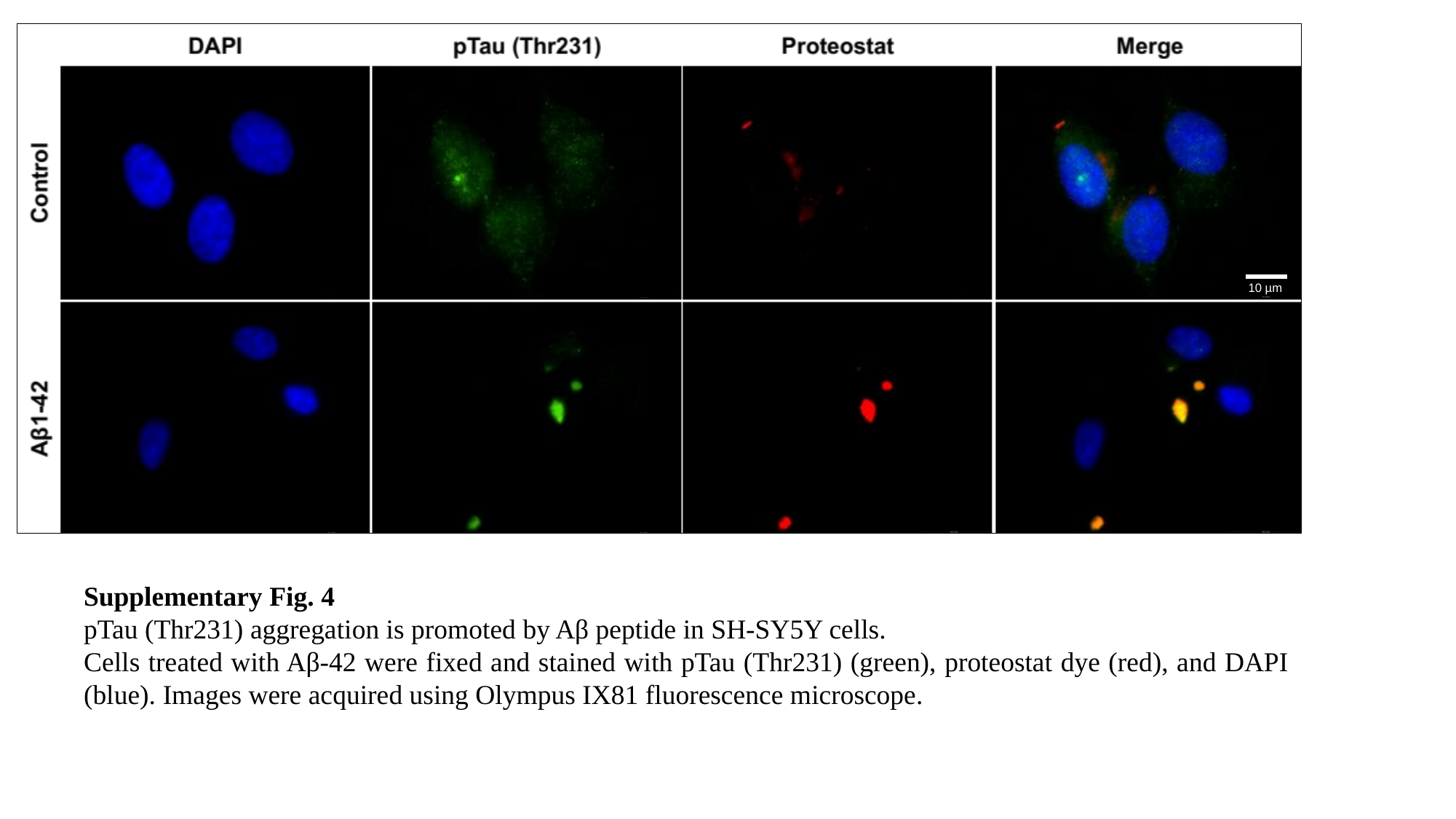

10 µm
Supplementary Fig. 4
pTau (Thr231) aggregation is promoted by Aβ peptide in SH-SY5Y cells.
Cells treated with Aβ-42 were fixed and stained with pTau (Thr231) (green), proteostat dye (red), and DAPI (blue). Images were acquired using Olympus IX81 fluorescence microscope.

## Slide 5
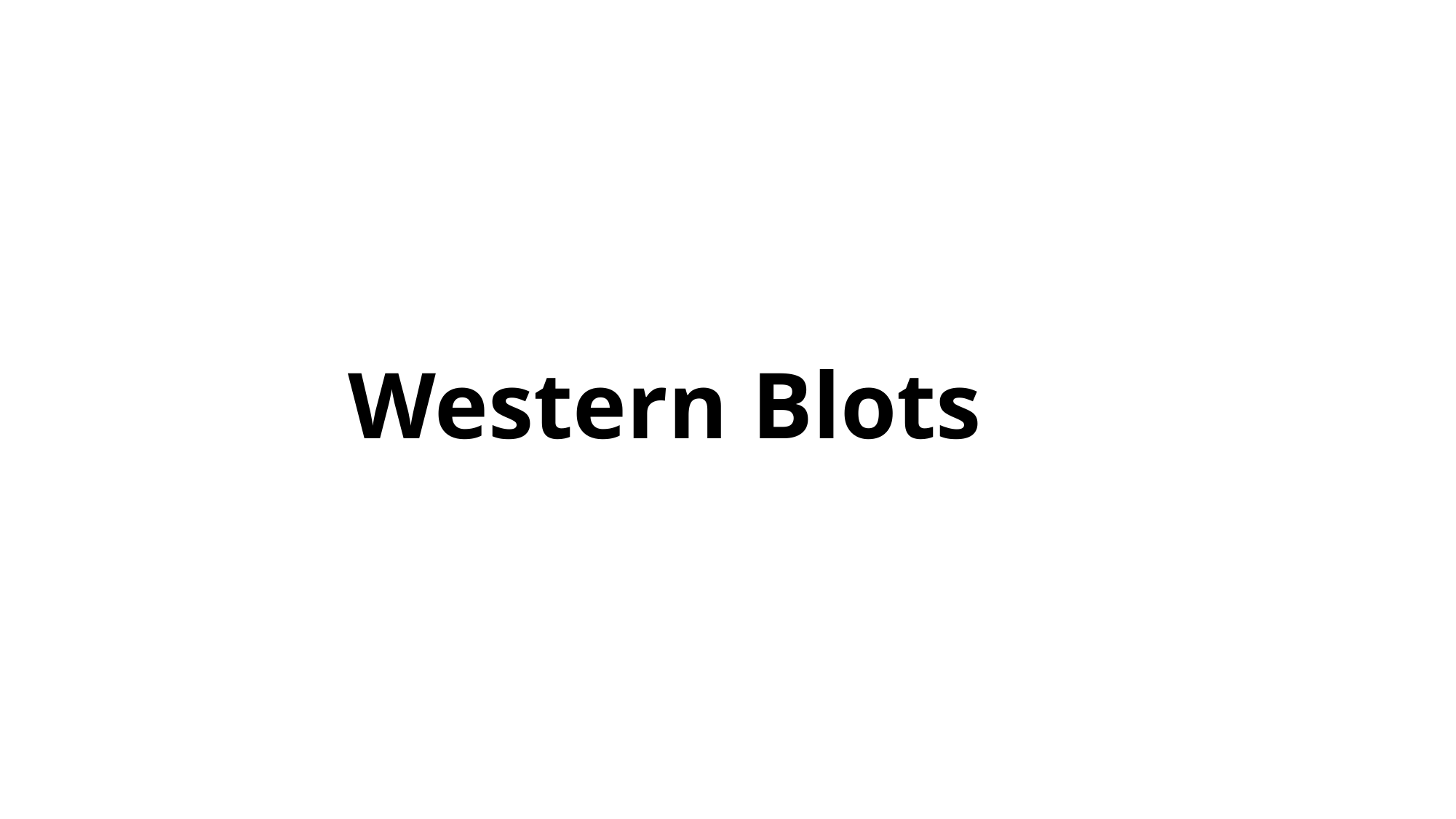

# Western Blots

## Slide 6
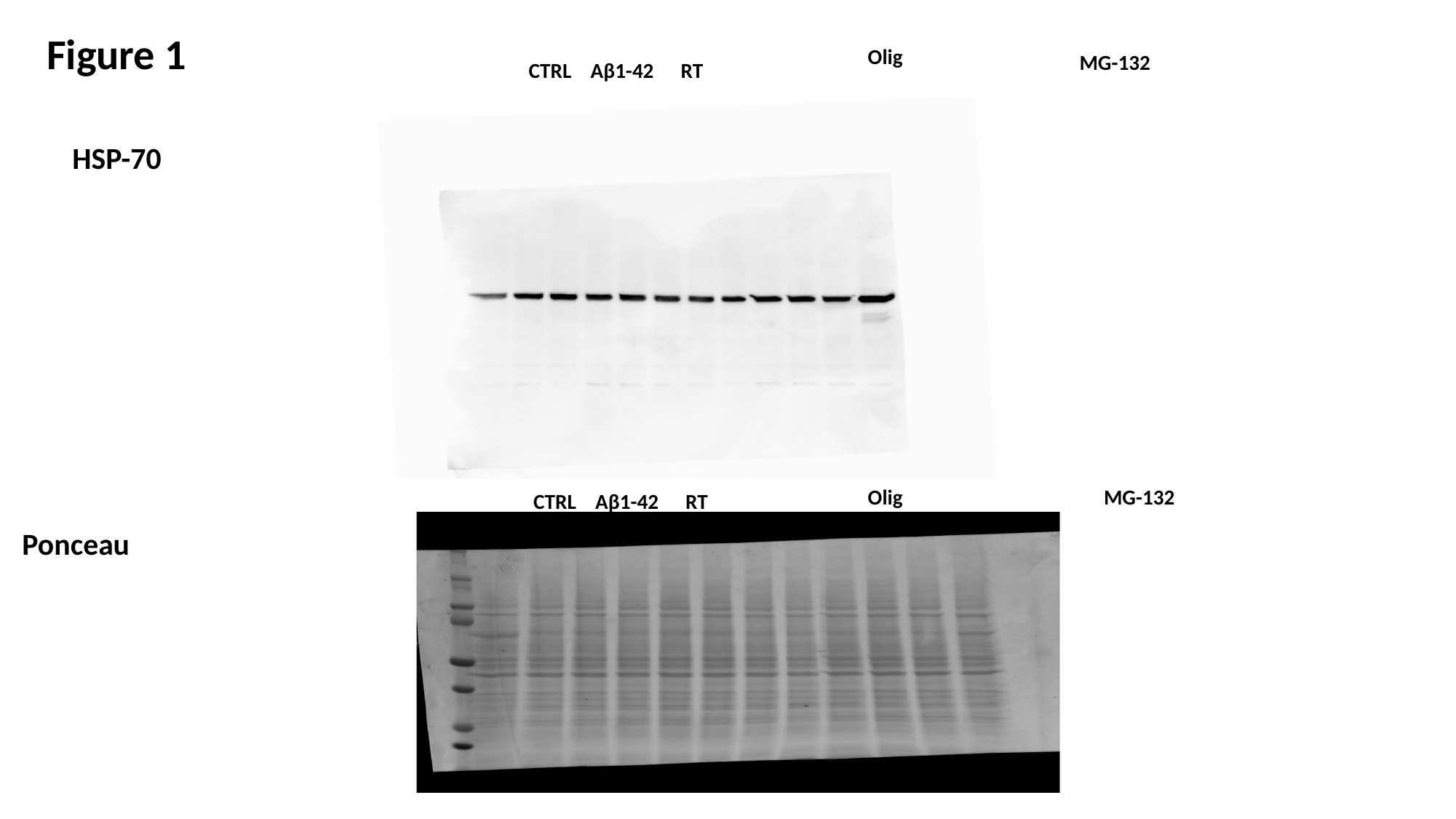

Figure 1
Olig
MG-132
CTRL
Aβ1-42
RT
HSP-70
Olig
MG-132
CTRL
Aβ1-42
RT
Ponceau

## Slide 7
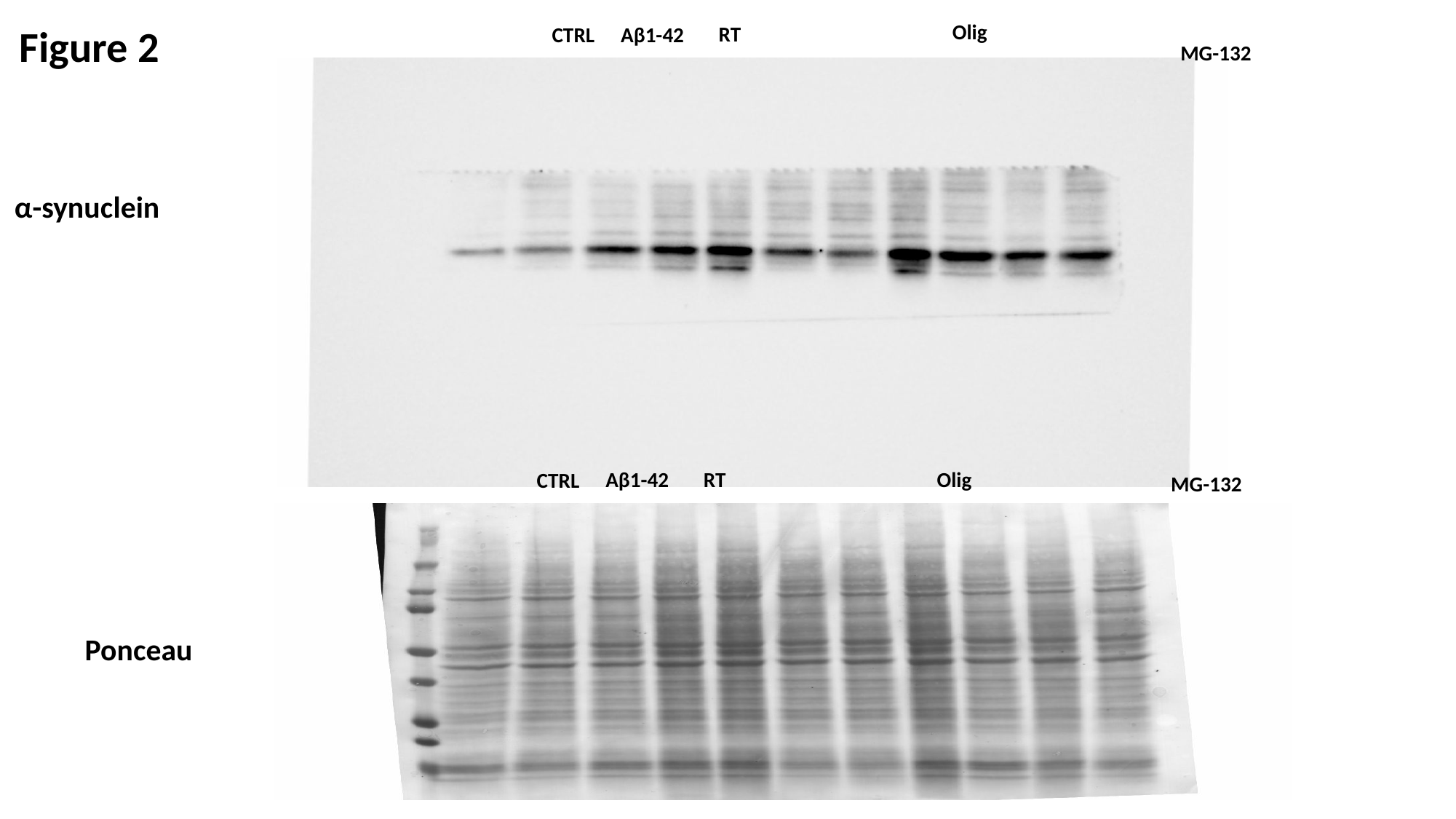

Olig
Figure 2
RT
Aβ1-42
CTRL
MG-132
α-synuclein
RT
Olig
Aβ1-42
CTRL
MG-132
Ponceau

## Slide 8
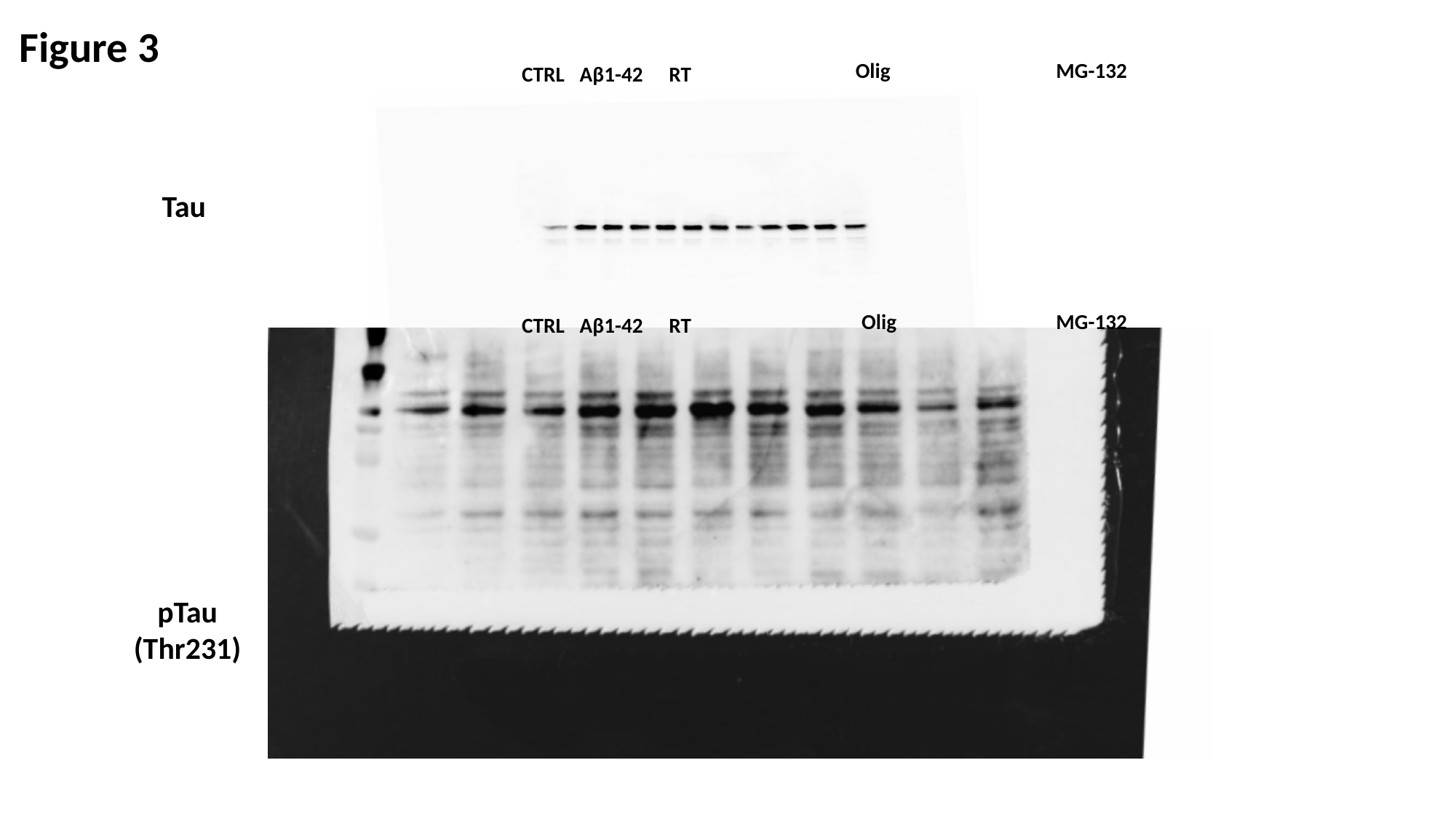

Figure 3
Olig
MG-132
CTRL
Aβ1-42
RT
Tau
Olig
MG-132
CTRL
Aβ1-42
RT
pTau (Thr231)

## Slide 9
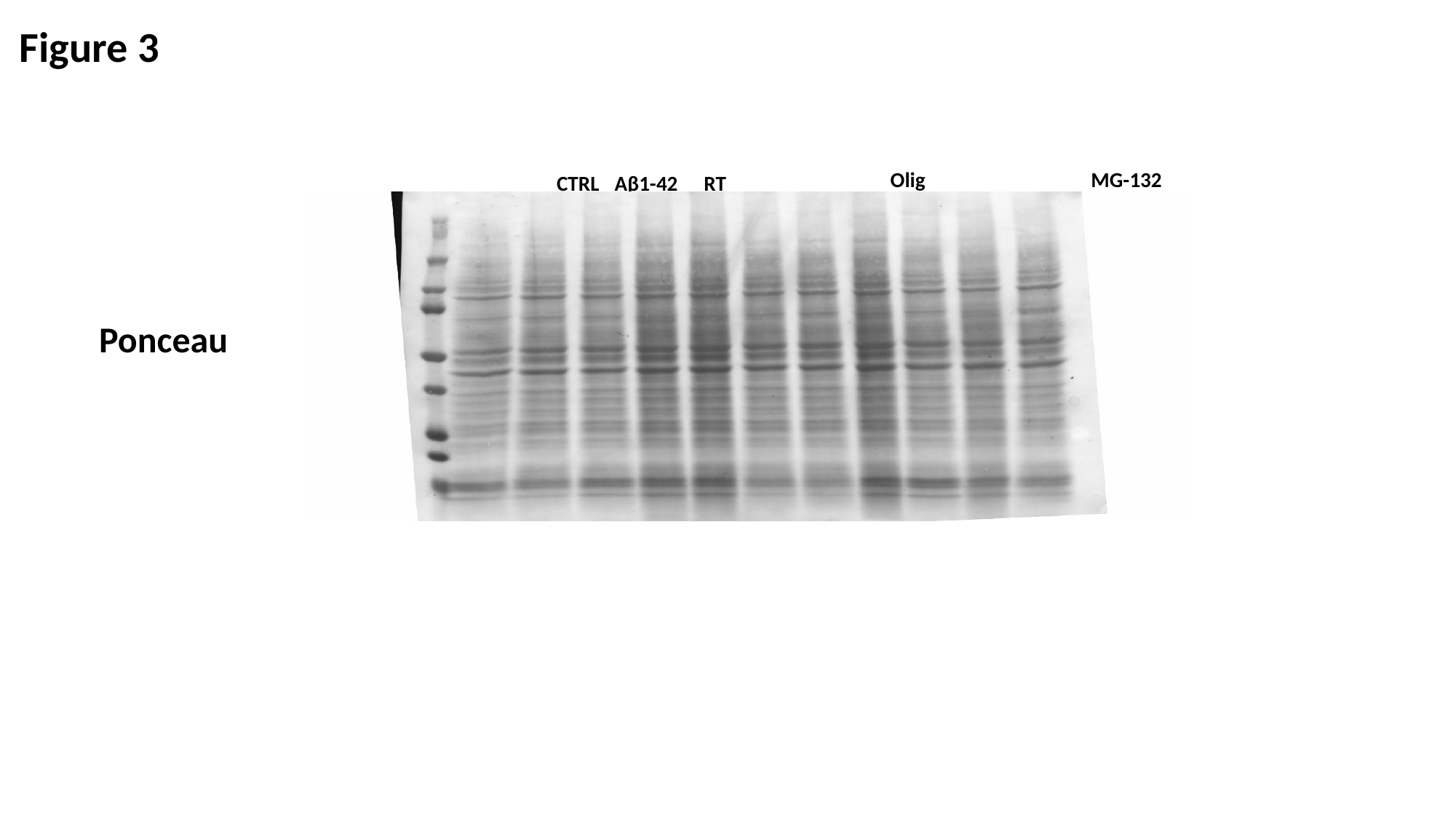

Figure 3
Olig
MG-132
CTRL
Aβ1-42
RT
Ponceau

## Slide 10
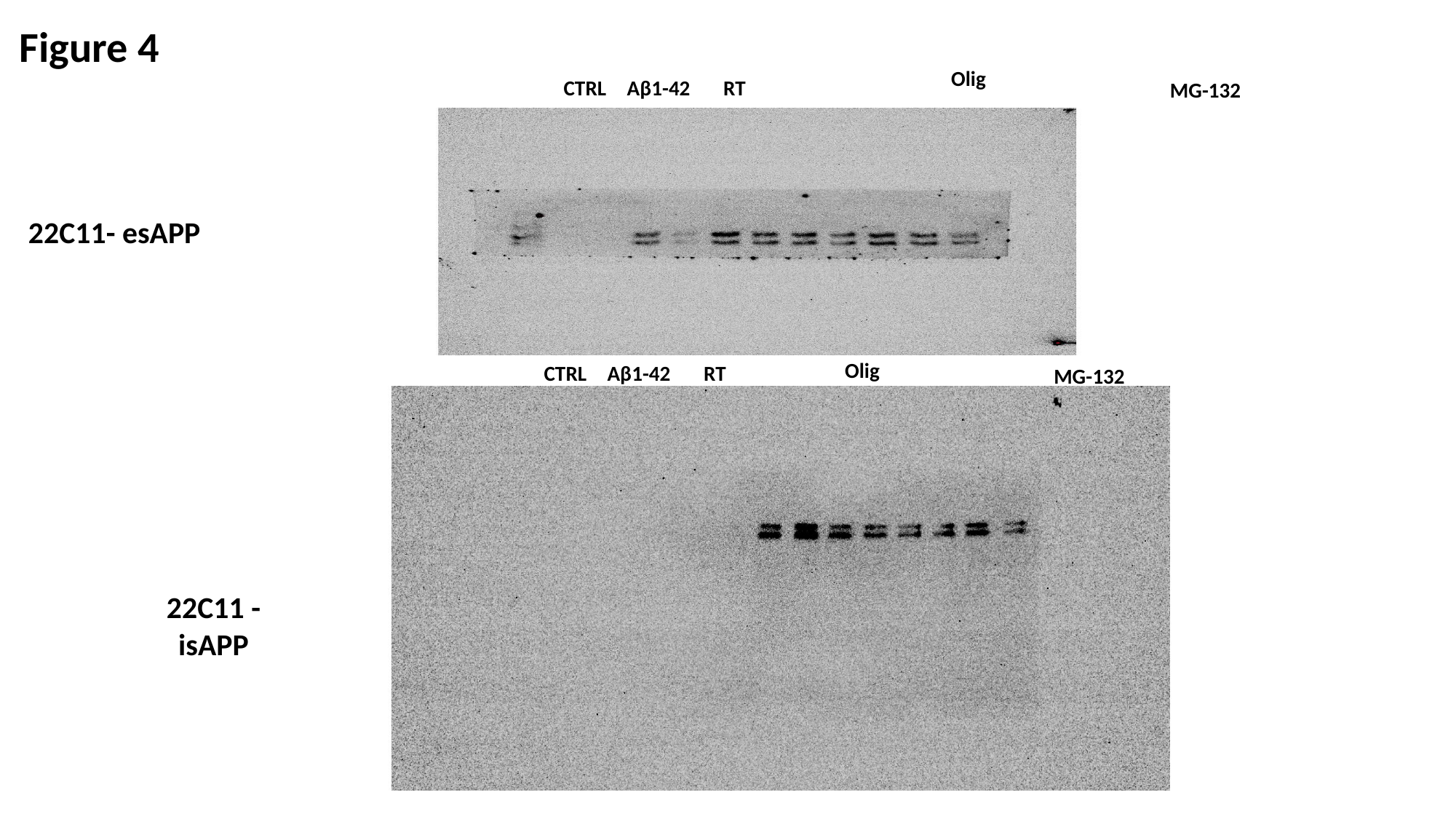

Figure 4
Olig
CTRL
Aβ1-42
RT
MG-132
22C11- esAPP
Olig
CTRL
Aβ1-42
RT
MG-132
22C11 - isAPP

## Slide 11
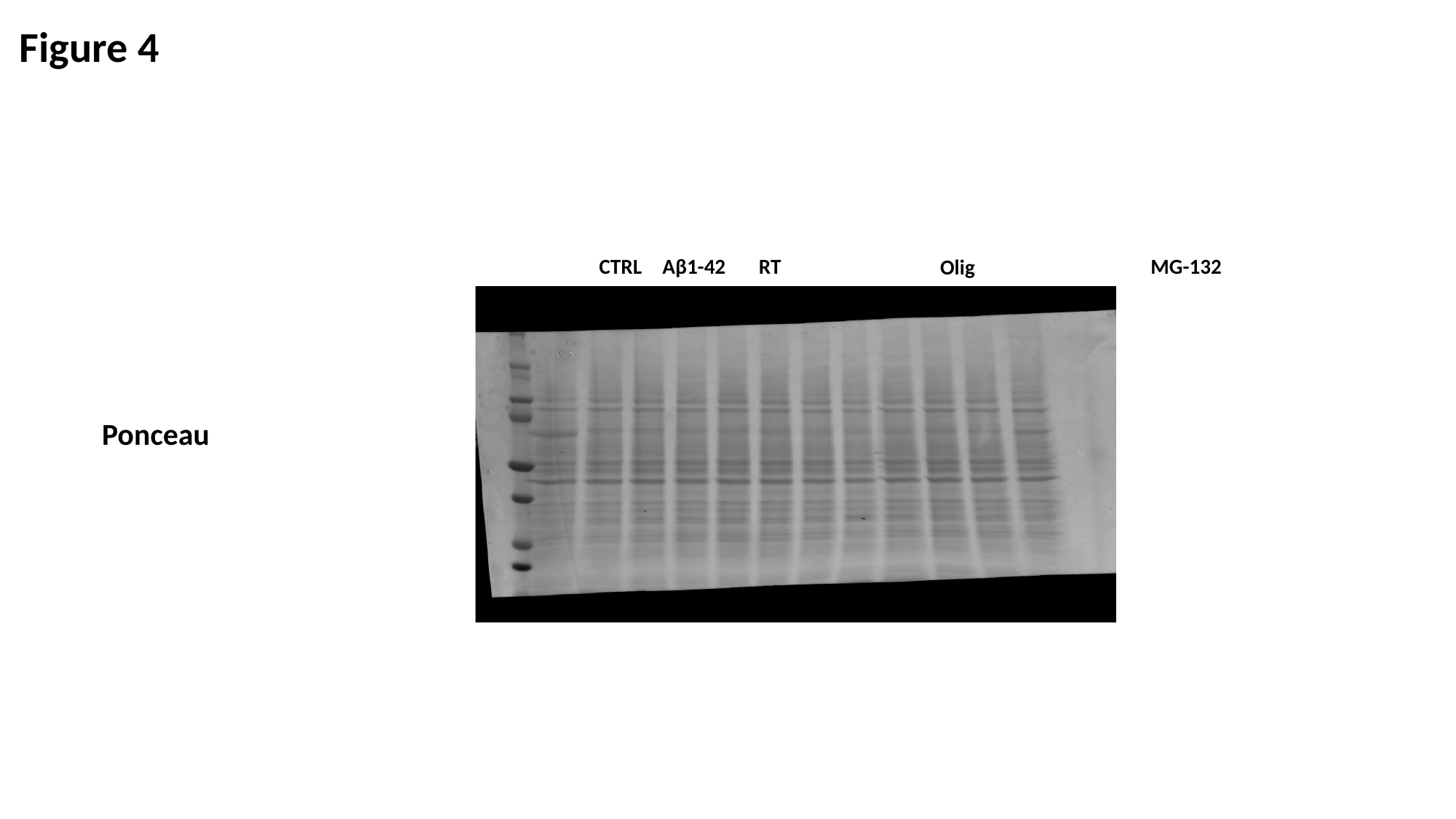

Figure 4
CTRL
Aβ1-42
RT
MG-132
Olig
Ponceau

## Slide 12
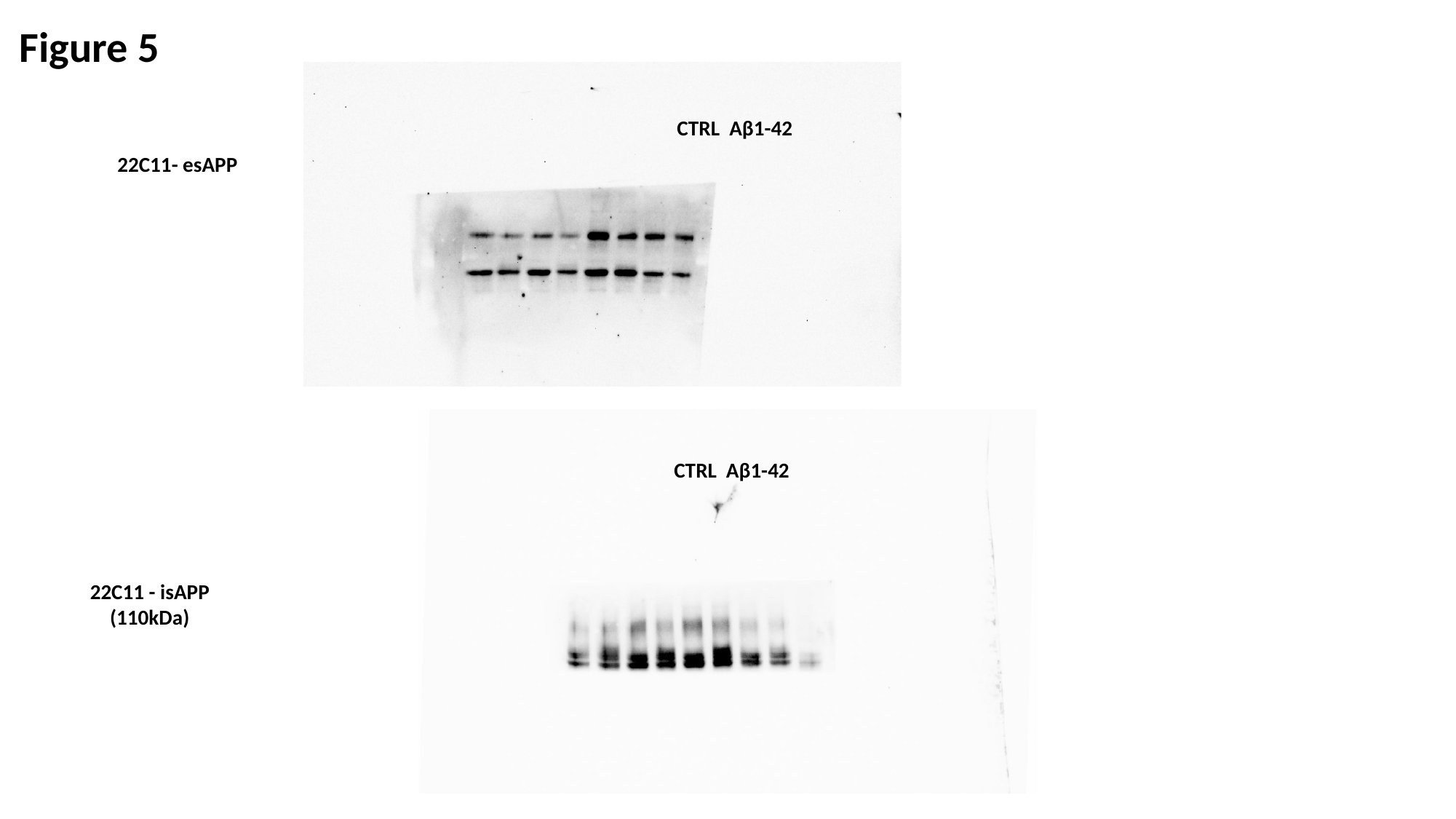

Figure 5
CTRL
Aβ1-42
22C11- esAPP
CTRL
Aβ1-42
22C11 - isAPP (110kDa)

## Slide 13
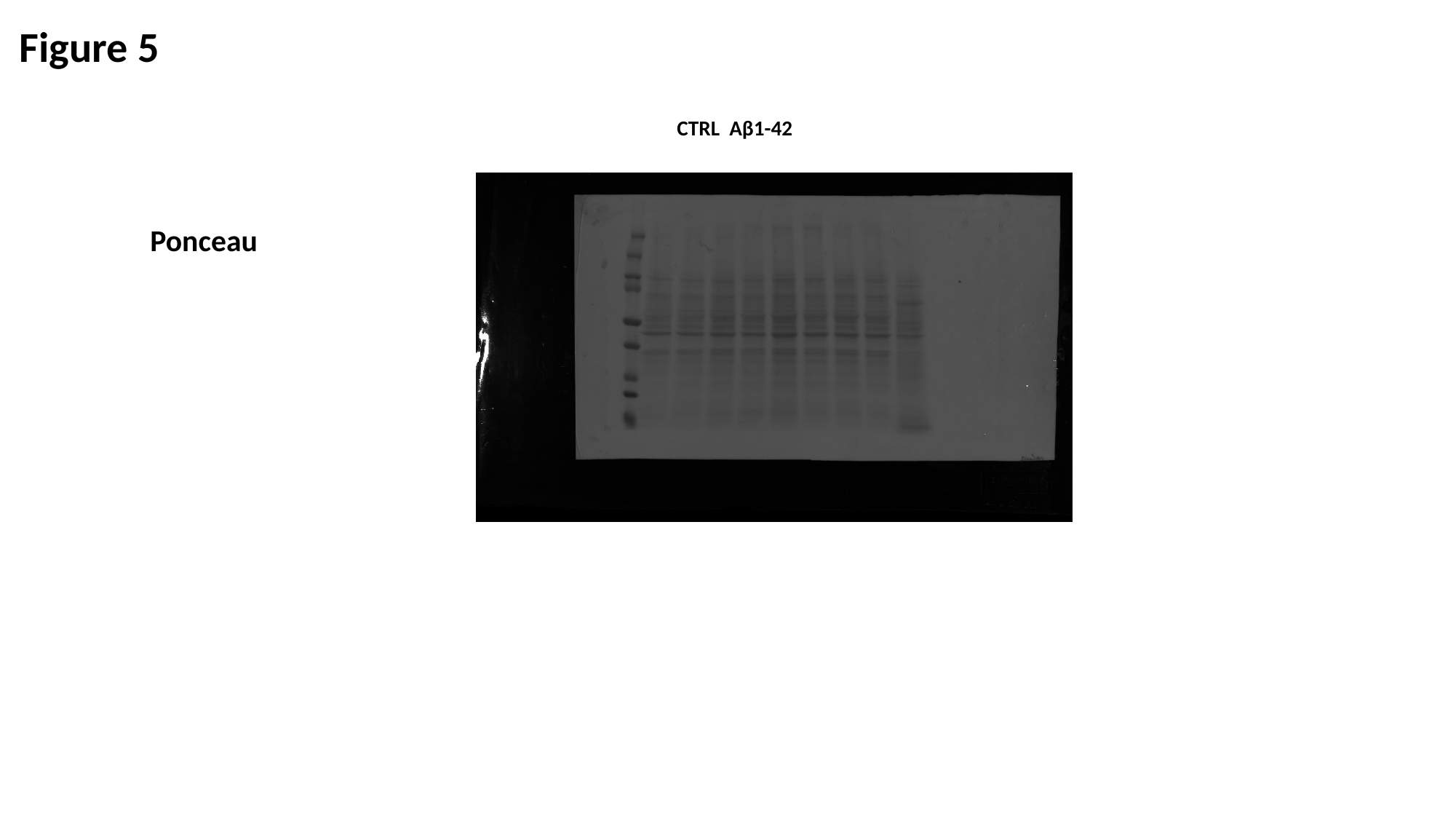

Figure 5
CTRL
Aβ1-42
Ponceau
